# An air-stable bisboron complex: a practical bidentate Lewis acid catalyst

**DOI:** 10.3762/bjoc.14.48

**Published:** 2018-03-13

**Authors:** Longcheng Hong, Sebastian Ahles, Andreas H Heindl, Gastelle Tiétcha, Andrey Petrov, Zhenpin Lu, Christian Logemann, Hermann A Wegner

**Affiliations:** 1 Institute of Organic Chemistry, Justus Liebig University Giessen, Heinrich-Buff-Ring 17, 35392 Giessen, Germany; 2 Current position: Department of Chemistry, University of Cambridge, Lensfield Road, Cambridge CB2 1EW, United Kingdom; 3 Institute of Inorganic Chemistry, University of Cologne, Greinstr. 4, 50939 Cologne, Germany

**Keywords:** air-stable catalyst, bidentate, bisboron, diazine, Diels–Alder, Lewis acid

## Abstract

We report an air-stable bisboron complex as an efficient catalyst for the inverse electron-demand Diels–Alder (IEDDA) reaction of 1,2-diazine as well as 1,2,4,5-tetrazine. Its stability towards air and moisture was demonstrated by NMR studies enabling its application in organic transformations without glovebox. A one-pot procedure for its synthesis was developed starting from 1,2-bis(trimethylsilyl)benzene greatly enhancing its practicality. Comparative reactions were carried out to evaluate its catalytic activity in IEDDA reactions of diazine including phthalazine as well as 1,2,4,5-tetrazine.

## Introduction

**Scheme 1:**
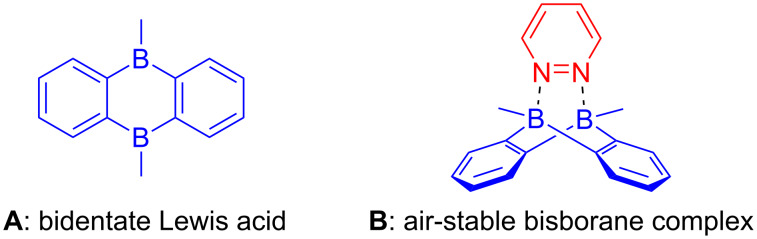
Bidentate bisborane Lewis acids.

The development of efficient and practical methods for synthesis is one of the prime objectives in chemistry. Especially transformations relying on new catalytic activation principles are of importance. In the past years, the inverse electron-demand Diels–Alder (IEDDA) reaction has been well established for the construction of cyclic frameworks, especially in the synthesis of natural products [1–2]. Aza-dienes have been utilized in IEDDA reactions for the construction of nitrogen-containing heterocyclic compounds [3–8]. Among them, 1,2-diazines are less used owing to their relative low reactivity as their LUMOs are rather high in energy [9–12]. In the past years, we demonstrated a bisboron bidentate Lewis acid (Scheme 1, **A**) as an efficient catalyst for the IEDDA reaction of 1,2-diazines to access 1,2-substituted aromatics [13–14]. Additionally, we could incorporate this reactivity in domino reactions combining the IEDDA step with rearrangements [15] or additional Diels–Alder reactions [16]. However, the methodology requires an air and moisture-sensitive bisboron catalyst. The preparation as well as the handling requires special equipment such as a glovebox, therefore, restricting its applicability [17]. To overcome this limitation we herein present a new catalyst (Scheme 1, **B**) with reasonable stability to air and moisture, which can be handled under ambient conditions and prepared with standard laboratory equipment.

## Results and Discussion

Generally, the reactivity of boron Lewis acids is attributed to the free vacant p-orbital of the boron atom leading to further transformations, such as decomposition via radicals (O_2_), reactions with nucleophiles (H_2_O) as well as the formations of adducts. From this perspective, a suitable Lewis base may form a Lewis complex and subsequently occupy the p-orbital of the boron atom. This may prevent the boron compound from decomposition as well as hydrolysis and provide a practical Lewis acid catalyst for organic reactions.

**Scheme 2:**
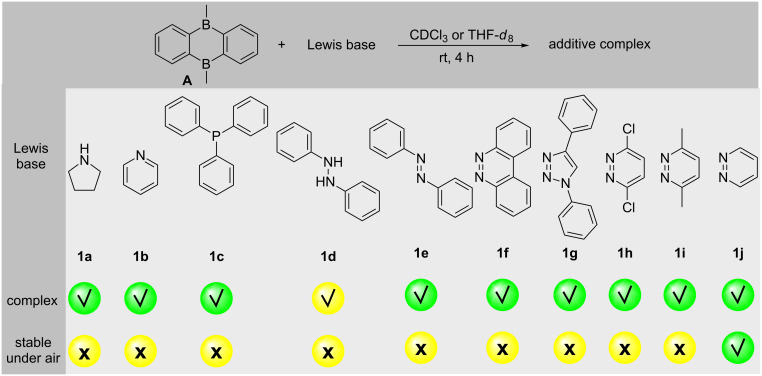
Complexation reaction of 5,10-dimethyl-5,10-dihydroboranthrene (**A**) with Lewis bases analyzed by NMR proton spectroscopy.

**Figure 1:**
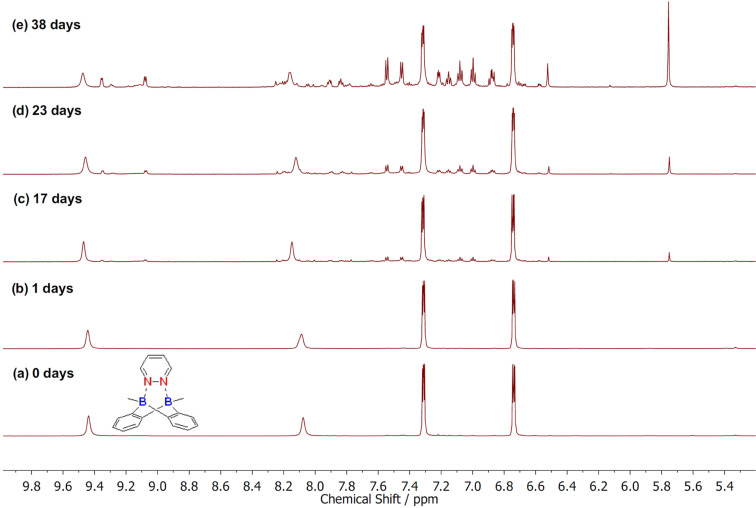
Time-dependent ^1^H NMR spectra of the air-exposed complex **B**.

To test the hypothesis, several Lewis bases were subjected to the coordination reaction with the bidentate bisboron catalyst, 5,10-dimethyl-5,10-dihydroboranthrene (**A** in Scheme 1), developed in our group [13]. A complexation was confirmed by a high field shift of the aromatic protons compared with the non-coordinated catalyst **A**. As shown in Scheme 2, besides 1,2-diphenylhydrazine (**1d**), most of the Lewis bases including monodentate **1a**–**1c** and bidentate Lewis bases **1e**–**1j** can coordinate with bisboron compound **A** as determined by NMR spectroscopy (see Supporting Information File 1). The stability was then evaluated exposing the resulting complexes to air. However, most adducts quickly decomposed under ambient conditions except the adduct **B** of pyridazine (**1j**). To further evaluate the stability of complex **B**, a time-dependent ^1^H NMR study was conducted. The first signs of decomposition appeared in the low field regions of the ^1^H NMR spectrum after 17 days (Figure 1c). These decomposition signals increased with ongoing air exposure (Figure 1c–1e). Nevertheless, there was a significant amount of complex **B** remaining even after 38 days of air exposure (Figure 1e).

### Optimized procedure for the synthesis of complex B

**Scheme 3:**
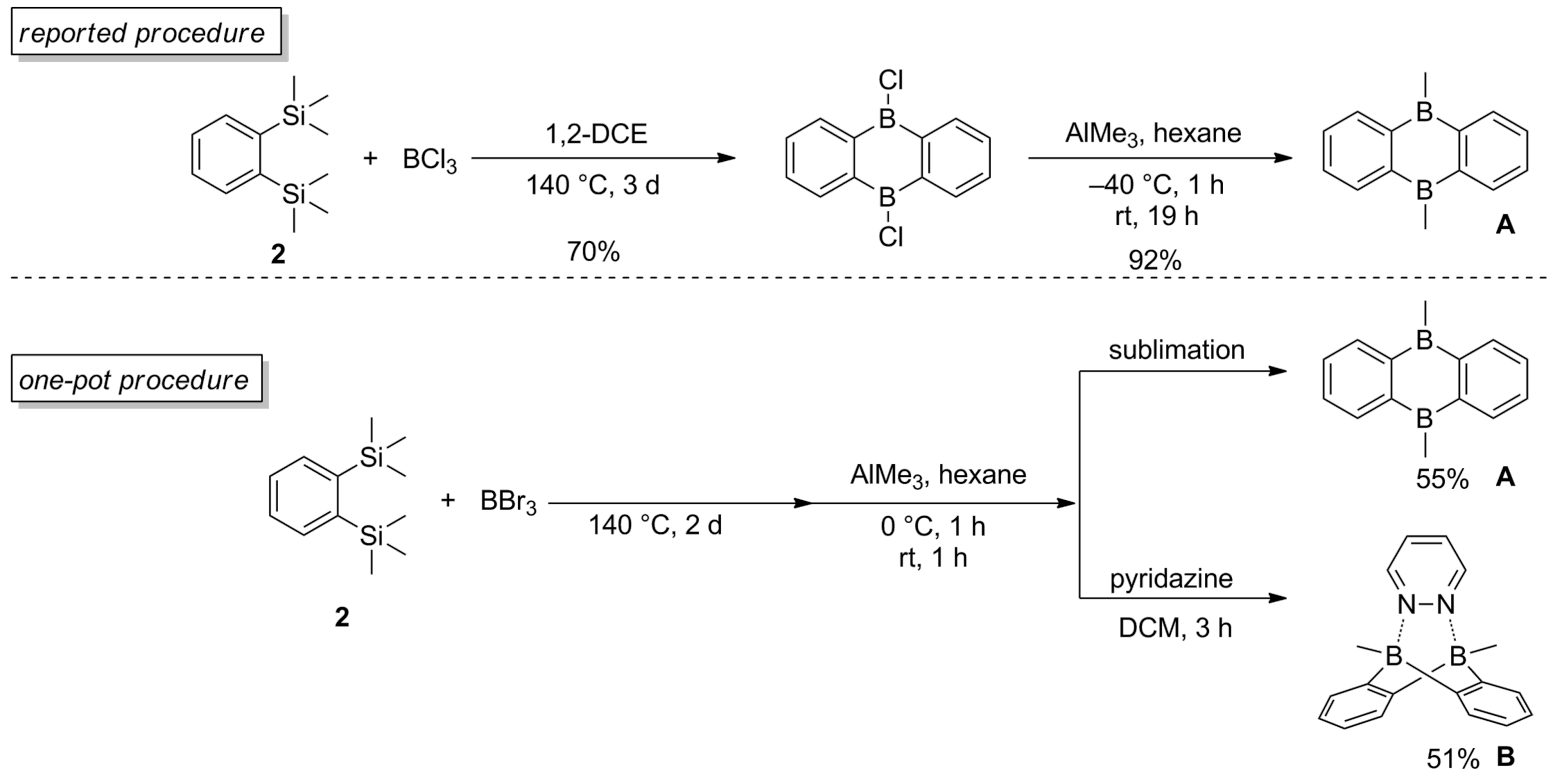
Synthetic procedures of bisboranes **A** and **B**.

The synthesis of bisboron compound **A** has been well described by Wagner’s group [18] as well as by our group [17]. A typical procedure for the synthesis of bisboron compound **A** via a dimerization requires a high reaction temperature (Scheme 3, top). Due to the use of volatile BCl_3_, the dimerization reaction is usually carried out in a sealed pressure tube. Using Schlenk technique, the procedure affords compound **A** in good yield. However, the bidentate bisboron Lewis acid **A**, which is also the boron source of complex **B**, is usually handled in a glovebox.

To facilitate the application of complex **B**, a new one-pot procedure was developed for its preparation starting from 1,2-bis(trimethylsilyl)benzene (**2**, Scheme 3, bottom). In the optimized procedure, BBr_3_ was used to replace BCl_3_ and the dimerization reaction can be carried out in a normal Schlenk tube without any solvent. The methylation reagent, AlMe_3_ can be added in situ followed by the complexation with pyridazine. The one-pot procedure is also applicable to the synthesis of compound **A**. In that case, after removal of all the volatile components under reduced pressure, a cooling finger can be installed on the Schlenk tube for sublimation affording compound **A** in 55% overall yield.

### X-ray crystal structure analysis

**Figure 2:**
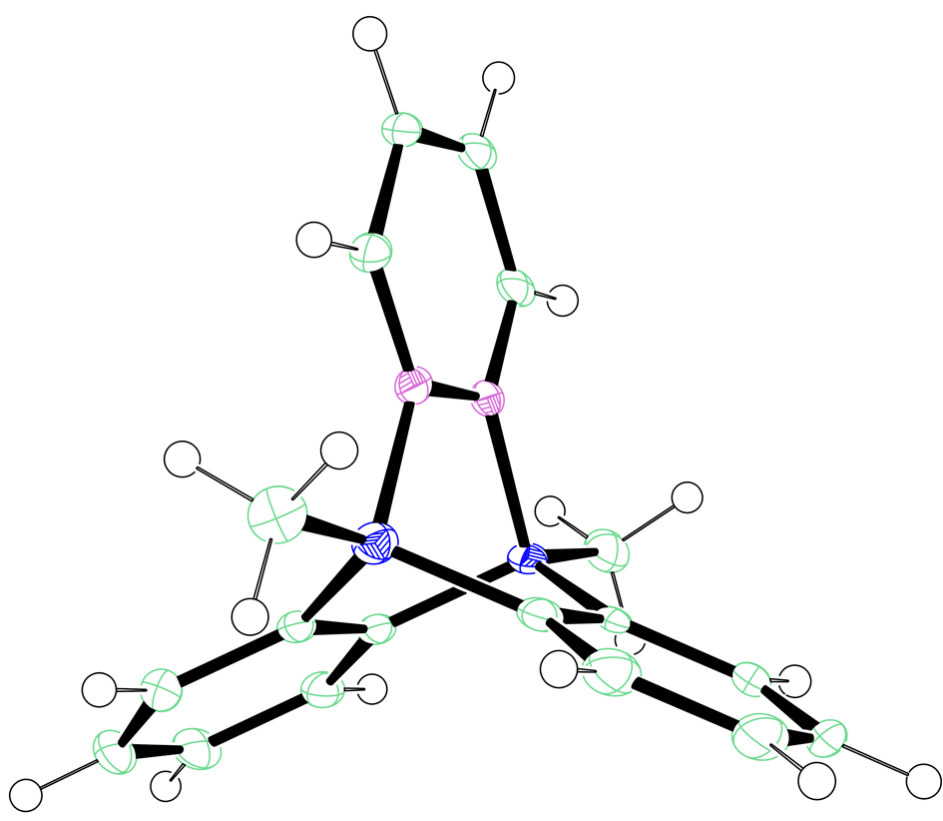
ORTEP drawing (50% probability) of complex **B**.

A single crystal of the air-stable Lewis acid **B** was obtained from EtOAc/BrPh 1:1 (v/v). The X-ray analysis revealed a triptycene-type arrangement (Figure 2) which was similar to a pyridazine complex of 9,10-dihydro-9,10-diboraanthracene reported by Wagner and co-workers [19] as well as the phthalazine complex of 5,10-dimethyl-5,10-dihydroboranthrene [17]. The ^11^B NMR spectrum of complex **B** showed one resonance at 2.4 ppm which also demonstrated the tetra-coordination of boron [20].

### UV–vis spectroscopy analysis

**Figure 3:**
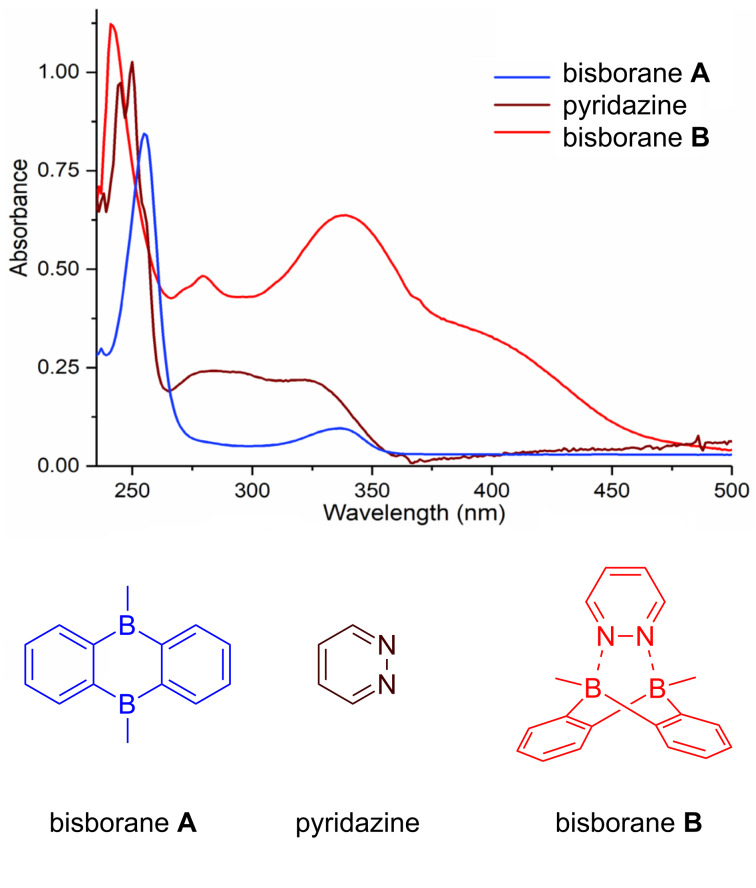
UV–vis spectrum of complex **B** was measured in CHCl_3_ and compared with pyridazine and bisborane **A** (concentrations: **B**: 4.52 × 10^−5^ mol/L, **A**: 4.52 × 10^−5^ mol/L, pyridazine: 3.68 × 10^−5^ mol/L).

The absorption properties of the air-stable complex **B** was investigated by UV–vis spectroscopy. As shown in Figure 3, there were three absorption bands at 241 nm, 279 nm, 339 nm, respectively, existing for complex **B**. A comparison of **A** with **B** revealed that the sharp absorption maximum at 255 nm of **A** underwent a blue shift after coordination with pyridazine, and an additional broad absorption at around 400 nm appeared.

### Catalytic properties

The application of the air-stable Lewis acid **B** for organic synthesis was then tested in several IEDDA reactions of phthalazine (**3**) catalyzed by **B** or **A**, respectively. As shown in Table 1, both, dihydrofuran (Table 1, entry 1) and enamines, generated in situ from aldehydes and amines (Table 1, entries 2–4), can take part in the IEDDA reaction. However, the yields were significantly lower in reactions catalyzed with **B** when compared to catalyst **A** (Table 1, entries 1–4). Nonetheless, when a more active dienophile 6-ethoxy-1-methyl-1,2,3,4-tetrahydropyridine (**4f**) was subjected to the IEDDA reaction of phthalazine (**3**), the yields of the substituted naphthalene **5e** were comparable between the two bidentate Lewis acid catalysts (Table 1, entry 5). The binding of pyridazine (**1j**) with bisboron compound **A**, seems to be too strong to engage a fast ligand exchange with phthalazine (**3**).

**Table 1:** Comparison of different IEDDA reactions of phthalazine (**3**) catalyzed by bisboron compounds **A** and **B**^a^.

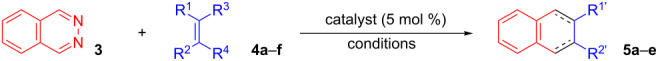			

entry	dienophile	product	yield

**1**^b^	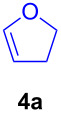	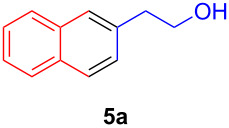	**A:** 43% **B:** 17%
**2**^c^	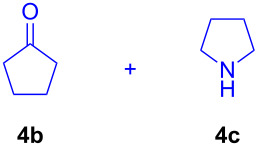	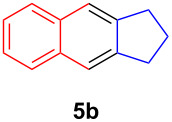	**A:** 52% **B:** 35%
**3**^d^	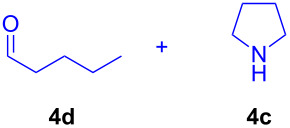	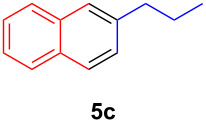	**A:** 12% **B:** 17%
**4**^e^	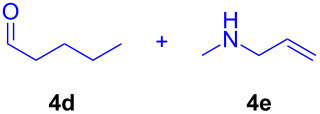	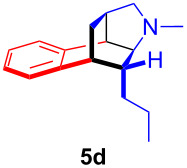	**A:** 93% **B:** 48%
**5**	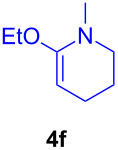	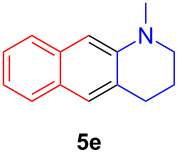	**A:** quant.^f^ **B:** 92%^g^

^a^General reaction conditions: phthalazine (1.00 equiv), catalyst **A** or **B** (5 mol %), dienophile (2.00 equiv; for enamines, generated in situ from aldehyde and amine), solvent (see Supporting Information File 1), and the reaction was carried out under N_2_ and stirred at given temperature; ^b^diglyme (0.6 mL), diisopropylethylamine (200 μL), 170 °C, 3 d; ^c^diglyme (0.45 mL), 55 °C, 60 h, work-up with *m*CPBA; ^d^THF (0.5 mL), 60 °C, 20 h; ^e^THF (1.5 mL), 60 °C, 15 h; ^f^diglyme (1.5 mL), 120 °C, 2.5 d; ^g^CF_3_Ph (1.5 mL), 100 °C, 19 h.

Recently we have demonstrated that catalyst **A** efficiently promotes the IEDDA reaction of 1,2,4,5-tetrazine (**6**) with 1,4-naphthaquinone (**7a**) [21]. Therefore, complex **B** was also tested as catalyst in the IEDDA reaction of 1,2,4,5-tetrazine (**6**) with 1,4-naphthaquinonic dienophiles **7a**–**7d**. As shown in Table 2, the product 2,3-diaza-9,10-anthraquinone (**8a**) was obtained in 93% yield catalyzed by **B** while the yield with **A** was only 76% (Table 2, entry 1). Furthermore, the air-stable bisboron complex **B** successfully catalyzed the reactions and allowed the synthesis of 2,3-diaza-5,12-naphthacenedione (**8b**), 6-methoxy-2,3-diaza-9,10-anthraquinone (**8c**), and 6,7-dimethoxy-2,3-diaza-9,10-anthraquinone (**8d**) in excellent yields (82%, 88%, 95%, respectively) supporting the practicality of this catalyst for IEDDA reactions (Table 2, entry 2–4).

**Table 2:** IEDDA reactions of 1,2,4,5-tetrazine catalyzed by bisboron catalysts **A** or **B**^a^.

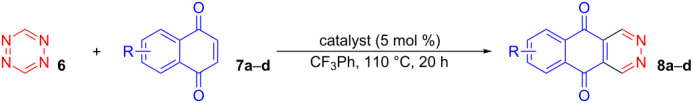			

entry	1,4-NQ	product	yield

**1**	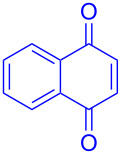 **7a**	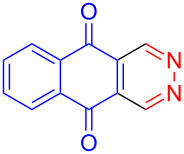 **8a**	**A:** 76% **B:** 93%
**2**	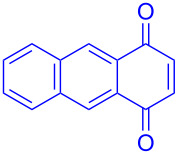 **7b**	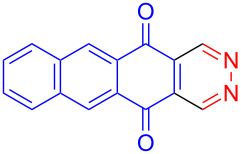 **8b**	**A:** 79% **B:** 82%
**3**	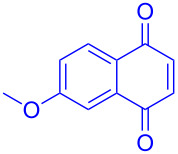 **7c**	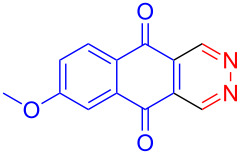 **8c**	**B:** 88%
**4**	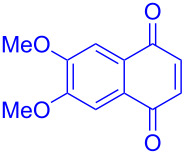 **7d**	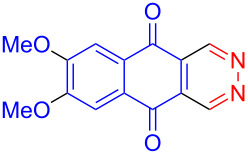 **8d**	**B:** 95%

^a^General reaction conditions: 1,4-naphthoquinone (1.00 equiv), catalyst **A** or **B** (5.00 mol %), 1,2,4,5-tetrazine (5.00 equiv) in CF_3_Ph (see Supporting Information File 1) was heated at 110 °C for 20 h under N_2_.

## Conclusion

In summary, we report an air-stable bidentate Lewis acid bisboron complex as an efficient catalyst for IEDDA reaction of 1,2-diazine as well as 1,2,4,5-tetrazine. Its stability towards air and moisture was demonstrated by NMR analysis and enables its application in organic synthesis without using a glovebox. A new one-pot procedure for its synthesis was developed starting from 1,2-bis(trimethylsilyl)benzene, which will greatly enhance its practicality. X-ray crystal structure analysis and UV–vis spectroscopy analysis were conducted. The tetra-coordination of the boron atoms and the highly symmetric molecular framework help to stabilize the adduct complex with respect to oxidation or hydrolysis. In addition comparative reactions were carried out and the results showed that the catalytic activity of the complex in IEDDA reactions depended on its performance in ligand exchange with the diazine substrates.

## Experimental

**Complexation of 5,10-dimethyl-5,10-dihydroboranthrene with Lewis bases:** In the glovebox, 5,10-dimethyl-5,10-dihydroboranthrene (**A**, 8.0 mg, 0.0392 mmol, 1.00 equiv) and Lewis base (for monodentate: 0.0784 mmol, 2.00 equiv; for bidentate: 0.0392 mmol, 1.00 equiv) were dissolved in 0.5 mL CDCl_3_ in an NMR tube (in case of 3,6-dimethylpyridazine, THF-*d*_8_ was used as solvent). The NMR tube was sealed and kept for 4 h and then monitored by ^1^H NMR spectroscopy.

**One-pot synthetic procedure of the bisboron–pyridazine complex B:** A Schlenk tube was charged with 1,2-bis(trimethylsilyl)benzene (1.78 g, 8.00 mmol, 1.00 equiv) and a stirring bar. Then, boron tribromide (4.21 g, 1.7 mL, 16.8 mmol, 2.10 equiv) was added slowly under N_2_ while stirring the reaction mixture. Afterward, the reaction mixture was stirred for 1 h at rt and stirring was continued at 140 °C for 2 d. After cooling the reaction mixture to rt, the excess boron tribromide was removed under reduced pressure connected to a cold trap. The Schlenk tube was filled again with N_2_ and *n*-hexane (degassed, 15.0 mL) was added. The tube was then placed in an ice bath to precipitate the intermediate product. The liquid was removed by a syringe under N_2_ to afford a pale brown solid. Then, the residue was washed with degassed *n*-hexane (2 × 10.0 mL) under N_2_. Additional *n*-hexane (degassed, 10.0 mL) was added and the mixture was stirred at 0 °C for several minutes. Then, trimethylaluminum (4.0 mL, 2.0 M in *n*-hexane, 8.00 mmol, 1.00 equiv) was added under N_2_. After 1 h, the reaction mixture was warmed to rt and then stirred for an additional 1 h. The volatile components of the reaction mixture were removed under reduced pressure connected to a cold trap. To the residue was then added CH_2_Cl_2_ (degassed, 10.0 mL) followed by pyridazine (320 mg, 4.00 mmol, 0.500 equiv) under N_2_. After 3 h of stirring, the reaction mixture was filtrated with a Büchner funnel and the solid was washed with DCM twice to afford a yellow solid (581 mg, 51% yield, purity: 85% calculated based on ^1^H NMR). [Column chromatography on silica gel (EtOAc/cyclohexane 4:1) yielded pure product as an orange solid with a decreased yield (284 mg, 25% yield)]. ^1^H NMR (400 MHz, THF-*d*_8_) δ 9.26 (br s, 2H), 7.69 (br s, 2H), 7.28 (dd, ^3^*J* = 5.2 Hz, 3.2 Hz, 4H), 6.71 (dd, ^3^*J* = 5.2 Hz, 3.2 Hz, 4H), 0.95 (s, 6H); ^13^C NMR (100 MHz, THF-*d*_8_) δ 147.2, 133.7, 128.1, 124.2 (C atoms next to boron are not observable due to quadrupole coupling); ^11^B NMR (128 MHz, THF-*d*_8_) δ 2.4 (s)M; mp 252–253 °C; HRMS–ESI (*m*/*z*): [M + Na]^+^ calcd for C_18_H_18_B_2_N_2,_ 307.1548; found, 307.1561; IR (neat): 3124, 3041, 2965, 2918, 2829, 1581, 1468, 1425, 1310, 1286, 1276, 1153, 1113, 997, 950, 889, 754, 713, 610, 575.

### IEDDA reactions catalyzed by the air-stable bidentate Lewis acid catalyst B

**General procedure A for IEDDA reactions of phthalazine:** In a Schlenk tube charged with a stirring bar, the air-stable bidentate Lewis acid catalyst **B** (5.00 mol %) and the stated solvent were added under N_2_. Then, the phthalazine (1.00 equiv), dienophile (2.00 equiv; for enamines, generated in situ from aldehyde and amine) were added subsequently. The reaction mixture was stirred at the given temperature. After the reaction was finished, the solvent was removed. The remaining residue was purified by flash column chromatography over SiO_2_ to obtain the product.

**General procedure B for IEDDA reactions of 1,2,4,5-tetrazine:** The air-stable bidentate Lewis acid catalyst **B** (25.0 µmol, 5.00 mol %) and 1,2,4,5-tetrazine (5.00 equiv) in CF_3_Ph (2.5 mL) were thoroughly stirred for several minutes. Then, 1,4-naphthoquinone (1.00 equiv) was added, the reaction mixture was heated at 110 °C for 20 h. The solvent, together with the excess of 1,2,4,5-tetrazine were distilled off from the resulting mixture in vacuo. The residue was purified by column chromatography over SiO_2_ (ethyl acetate/cyclohexane 1:1) to obtain the product.

## Supporting Information

File 1 Detailed experimental procedures, copies of ^1^H and ^13^C NMR spectra, UV–vis spectra as well as the X-ray crystallography.

File 2 CIF of bisborane complex **B**.
