# Supplementary material for: An air-stable bisboron complex: a practical bidentate Lewis acid catalyst
Source: Beilstein J Org Chem. 2018 Mar 13;14:618–25. doi: 10.3762/bjoc.14.48 (PMC5870160; doi:10.3762/bjoc.14.48)
Supplement: File 1 — Detailed experimental procedures, copies of 1H and 13C NMR spectra, UV–vis spectra as well as the X-ray crystallography. [file Beilstein_J_Org_Chem-14-618-s001.pdf]

Supporting Information
for
An air-stable bisboron complex: a practical bidentate Lewis acid catalyst

Longcheng Hong[1], Sebastian Ahles[1], Andreas H. Heindl[1], Gastelle Tiétcha[1], Andrey Petrov[1], Zhenpin Lu[1,2], Christian Logemann[3] and Hermann A. Wegner[*1]

Address: [1]Institute of Organic Chemistry, Justus Liebig University Giessen Heinrich-Buff-Ring 17, 35392 Giessen, Germany, [2]Current position: Department of Chemistry, University of Cambridge, Lensfield Road, Cambridge, CB2 1EW, United Kingdom and [3]Institute of Inorganic Chemistry, University of Cologne, Greinstr. 4, 50939 Cologne, Germany

Email: Hermann A. Wegner - Hermann.A.Wegner@org.Chemie.uni-giessen.de
*Corresponding author

Detailed experimental procedures, copies of ^{1}H and ^{13}C NMR spectra, UV–vis spectra as well as the X-ray crystallography

Table of contents

1. General information
2. Complexation experiments of 5,10-dimethyl-5,10-dihydroboranthrene with Lewis bases
3. One-pot synthetic procedure of the bisborane-pyridazine complex
4. IEDDA reactions catalyzed by the air-stable bidentate Lewis acid catalyst **B**
5. UV–vis spectra
6. X-ray crystallography
7. References

General information

All solvents were purchased from Fisher Scientific, Sigma-Aldrich or Acros and were used as received. Amines were distilled over calcium hydride before used. Aldehydes were distilled and stored over 4 Å molecular sieves. Technical grade solvents for extraction and column chromatography were bulb-to-bulb distilled prior to usage. Air-sensitive reactions were set up using dry glassware in a glovebox, using Schlenk technique. ^{1}H and ^{13}C NMR experiments were performed at 25 °C on a Bruker DPX-NMR (400 MHz, 600 MHz) at 25 °C unless otherwise stated. Chemical shifts are reported in parts per million (ppm) related to solvent peak, coupling constants (J) are reported in hertz (Hz). NMR solvents were obtained from Cambridge Isotope Laboratories, Inc. (Andover, MA, USA) or Deutero GmbH. The multiplicities are written as s = singlet, d = doublet, t = triplet, m = multiplet and their combinations, such as dd = doublet of a doublet. Multiplets are reported as a span of their middle. Thin layer chromatography (TLC) was carried out on silica gel 60 F_{254} glass plates with a 0.25 mm layer or Polygram® Alox N/UV_{254} with a 0.2 mm coating and detected with a CAMAG UV Cabinet dual wavelength, 254/366 nm. Column chromatography was performed using silica gel 60 (0.040–0.063 mm). High resolution mass spectrometry (HRMS) was determined with a Thermo Scientific LTQ FT Ultra spectrometer (ESI) using methanolic solutions of the respective compounds or a Finnigan MAT95 sectorfield spectrometer (EI). The UV–vis spectra were recorded with a JASCO V-670 spectrophotometer (spectral range: 190–2500 nm, resolution ≥ 0.1 nm). For measurement of melting points, a Krüss KSP1N capillary

melting-point apparatus was utilized using a heating rate of 1 °C min^{-1}. All melting points were determined three times and are uncorrected. IR spectra were recorded on a Bruker IFS25 spectrometer using the attenuated total reflectance (ATR) method.

The bidentate bisborane Lewis acid catalyst 5,10-dimethyl-5,10-dihydrodiboraanthracene (**A**) was prepared according to the method described in the literature [1]. Pyrrolidine (**1a**), pyridine (**1b**), triphenylphosphane (**1c**), 1,2-diphenylhydrazine (**1d**), azobenzene (**1e**), benzo[*c*]cinnoline (**1f**), 3,6-dichloropyridazine (**1h**), pyridazine (**1j**), 2,3-dihydrofuran (**4a**), cyclopentanone (**4b**), pentanal (**4d**), *N*-methyl allylamine (**4e**), phthalazine (**3**), and 1,4-naphthaquinone (**7a**) are commercially available. 1,4-Diphenyl-1,2,3-triazole [2] (**1g**), 3,6-dimethylpyridazine [3] (**1i**), 6-ethoxy-1-methyl-1,2,3,4-tetrahydropyridine [1] (**4f**), anthracene-1,4-dione [4] (**7b**), 6-methoxy-1,4-naphthaquinone [5] (**7c**), and 6,7-dimethoxy-1,4-naphthaquinone [5] (**7d**) were prepared according to literature methods. Also 1,2,4,5-tetrazine was prepared according to literature procedure [6] and was stored in a refrigerator below −18 °C after additional purification by sublimation.

Complexation of 5,10-dimethyl-5,10-dihydroboranthrene with Lewis bases

^{1}H NMR Spectrum of the complexation with pyrrolidine in $CDCl_3$ (**1a**)

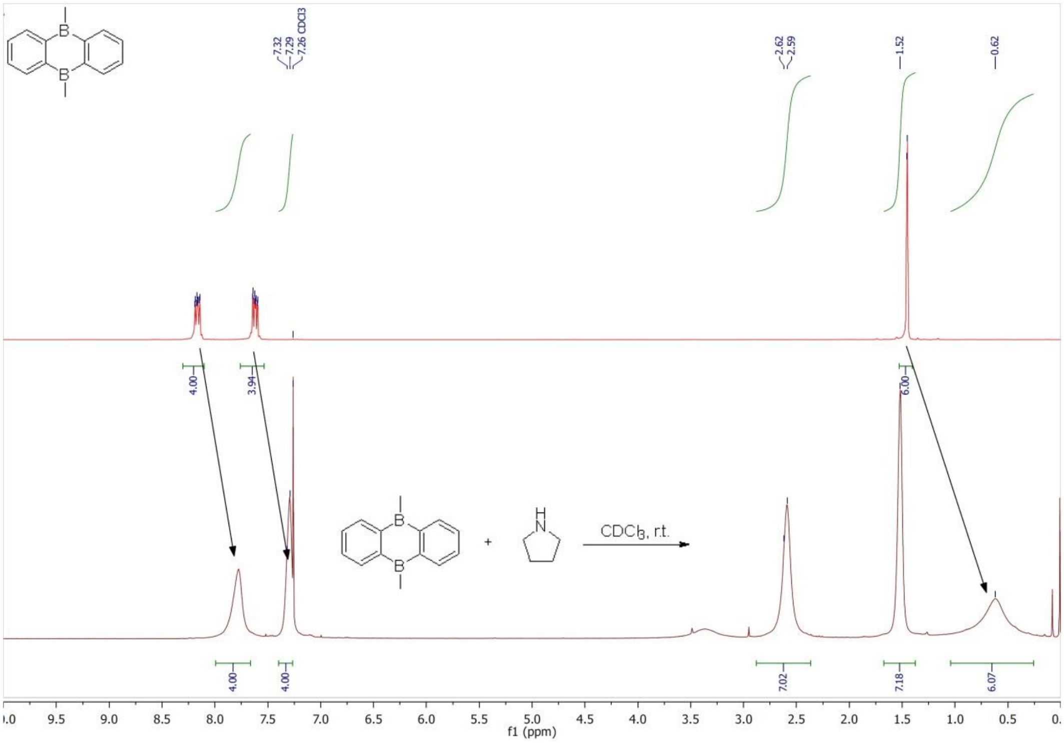

^{1}H NMR Spectrum of the complexation with pyridine in $CDCl_3$ (**1b**)

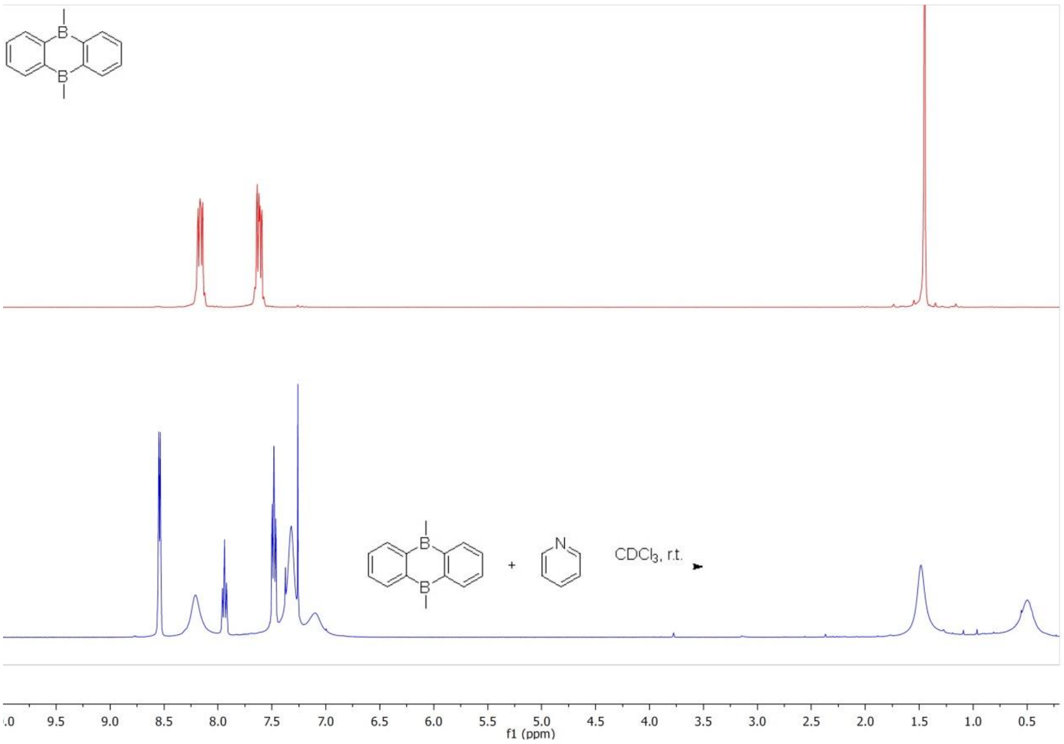

^{1}H NMR Spectrum of the complexation with triphenylphosphine in $CDCl_3$ (**1c**)

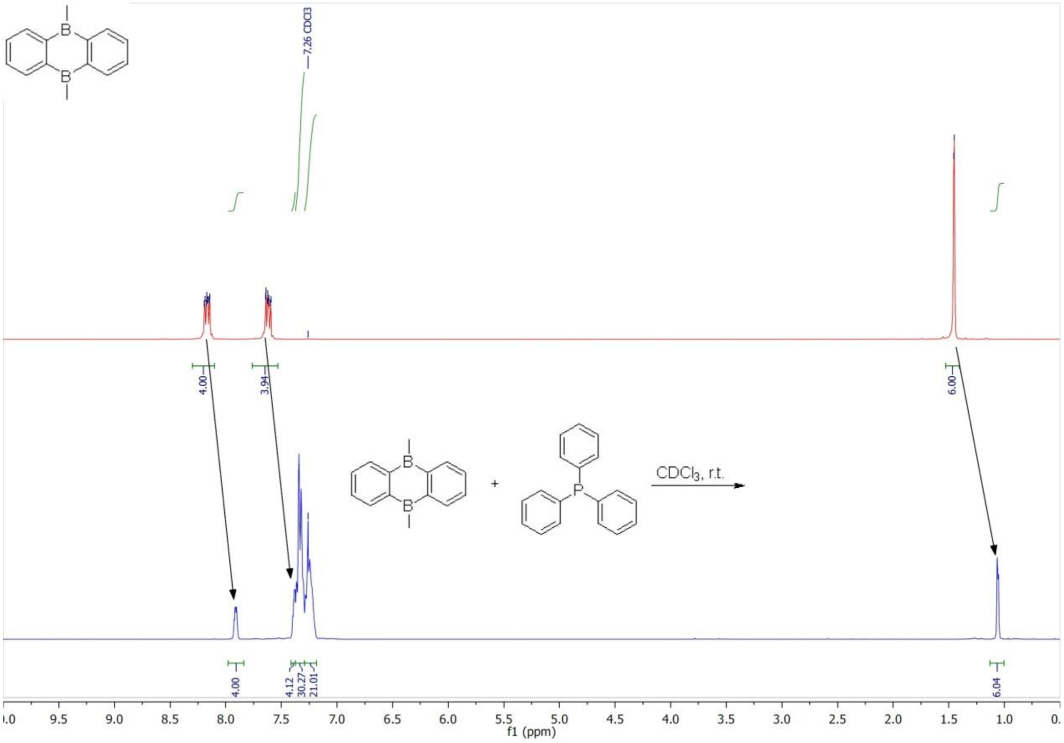

^{1}H NMR Spectrum of the complexation with 1,2-diphenylhydrazine in $CDCl_3$ (**1d**)

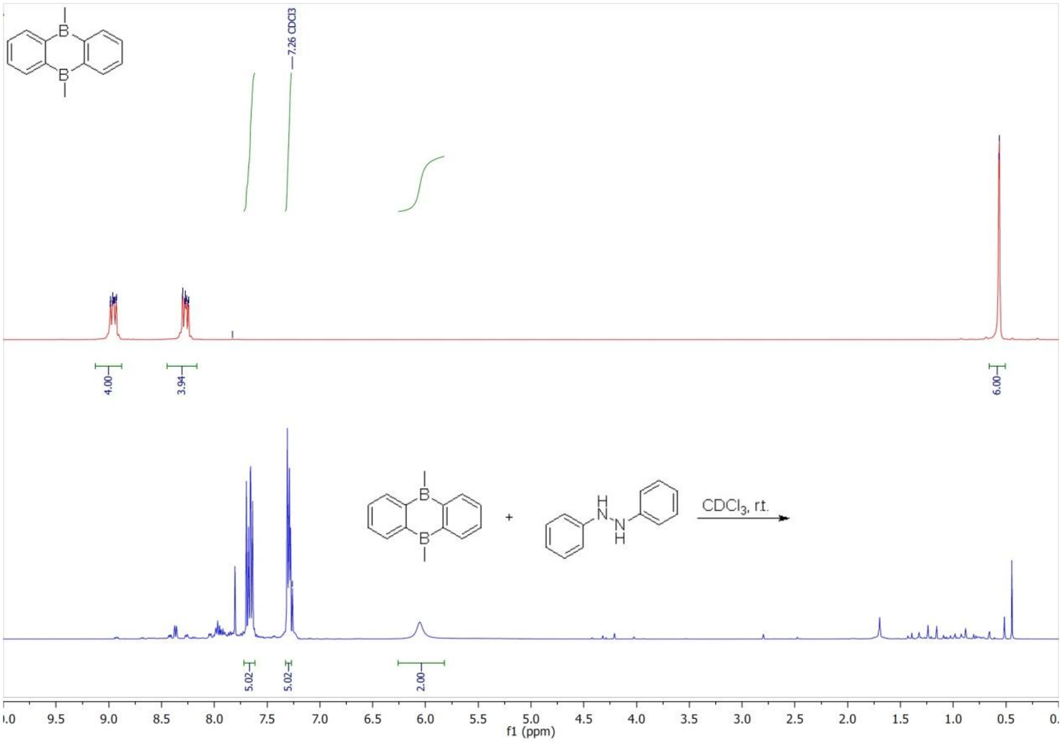

^{1}H NMR Spectrum of the complexation with azobenzene in $CDCl_3$ (**1e**)

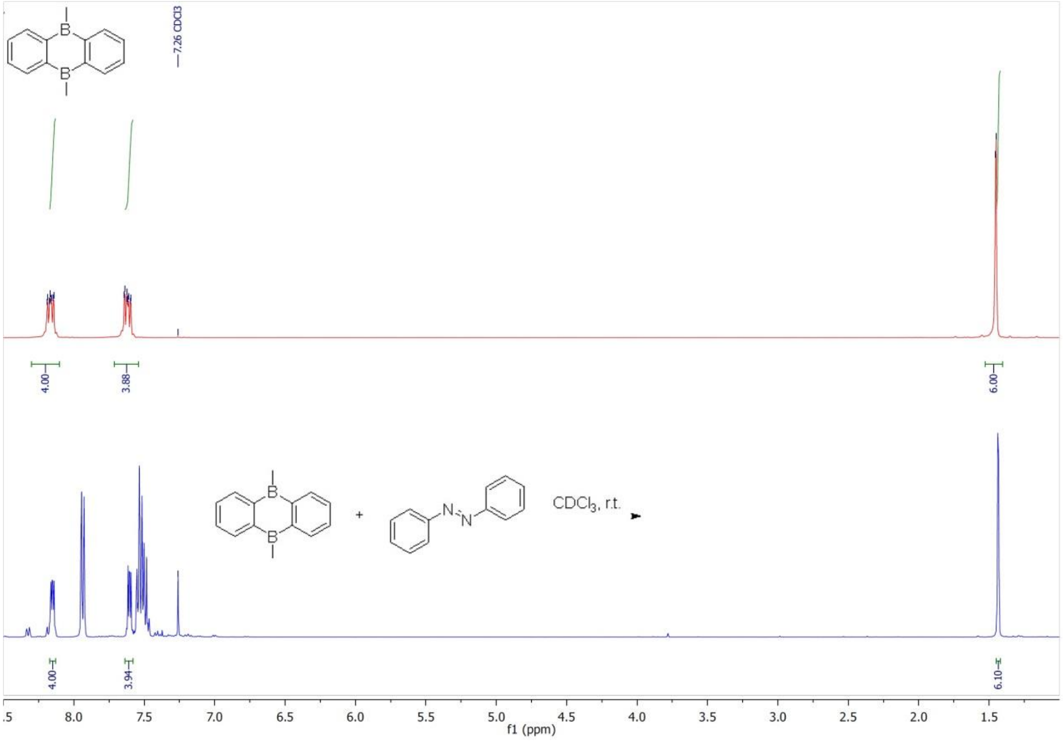

^{1}H NMR Spectrum of the complexation with benzo[*c*]cinnoline in d_8-THF (**1f**)

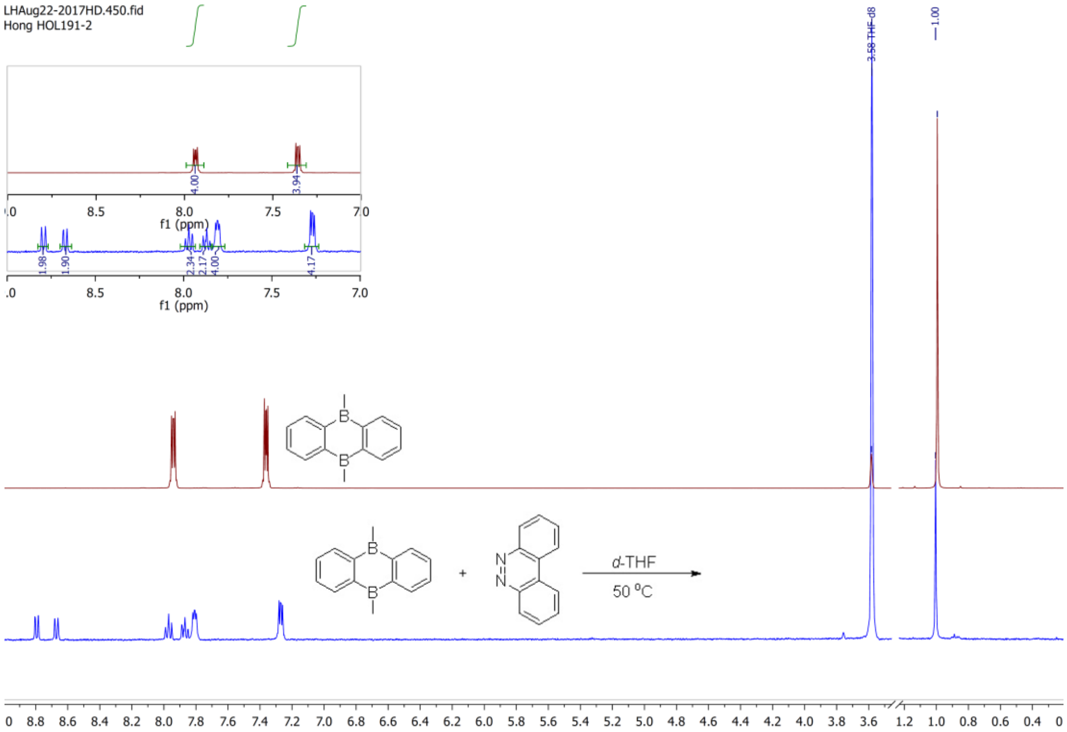

^{1}H NMR Spectrum of the complexation with 1,4-diphenyl-1,2,3-triazole in $CDCl_3$ (**1g**)

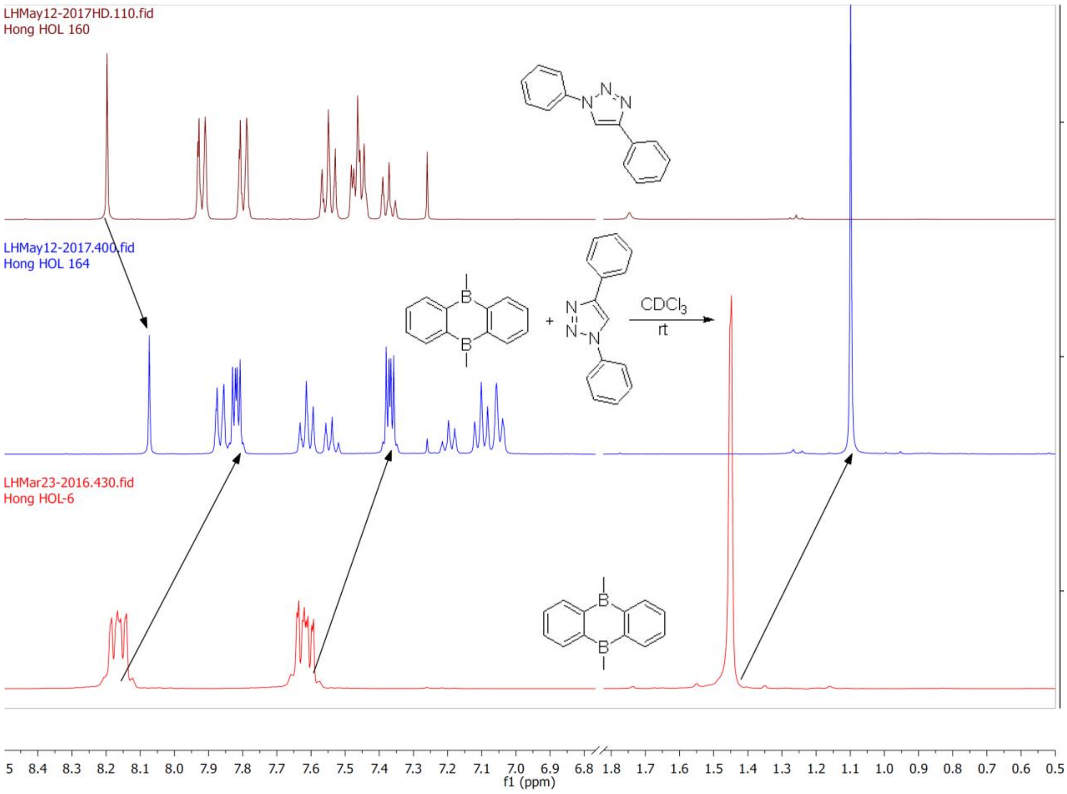

^{1}H NMR Spectrum of the complexation with 3,6-dichloro-pyridazine in $CDCl_3$ (**1h**)

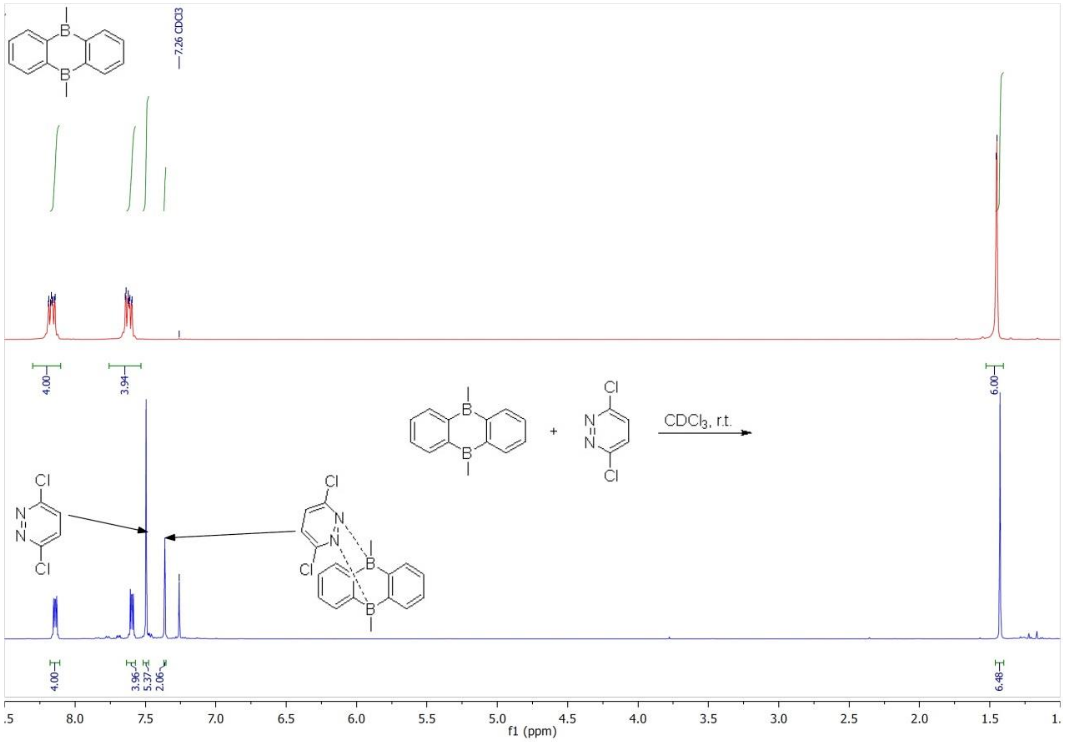

^{1}H NMR Spectrum of the complexation with 3,6-dimethyl-pyridazine in d_8-THF (**1i**)

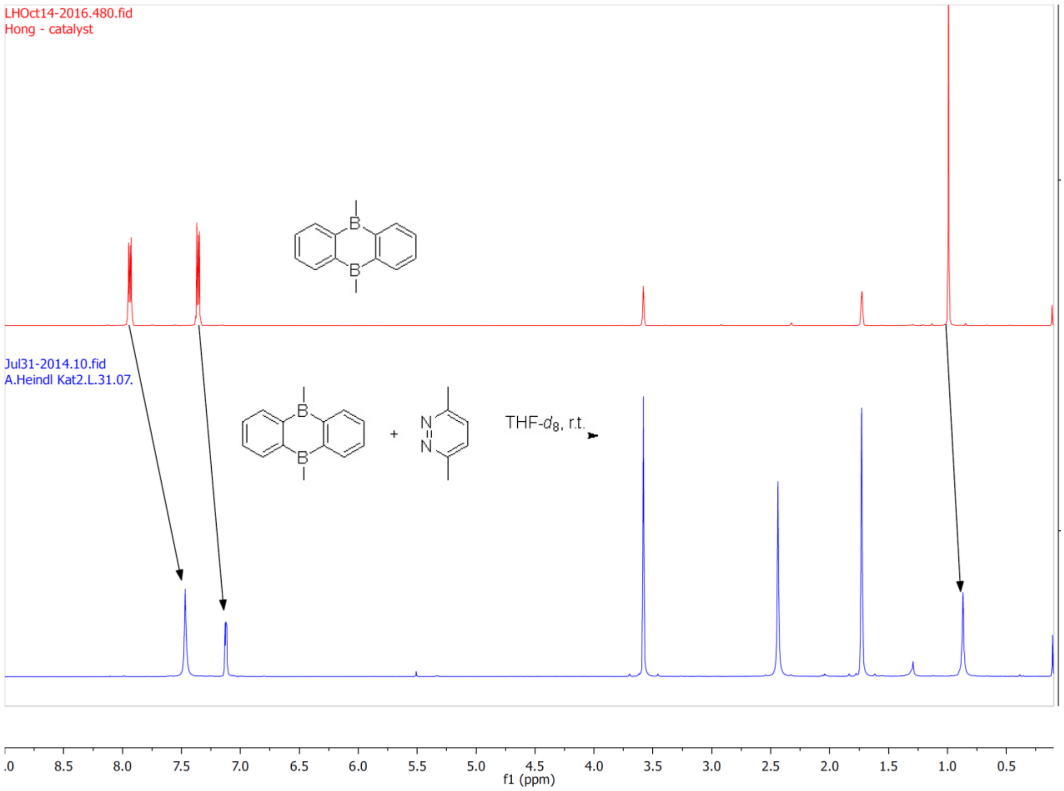

^{1}H NMR Spectrum of the complexation with pyridazine in $CDCl_3$ (**1j**)

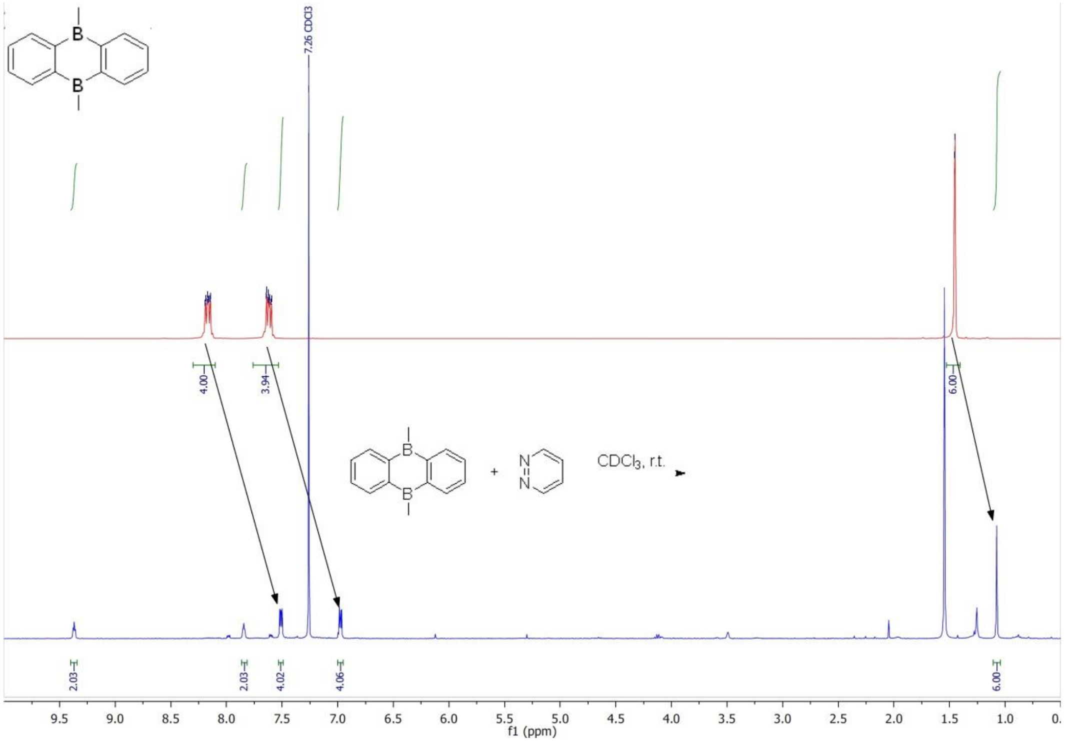

One-pot synthetic procedure of the bisborane/pyridazine complex

^{1}H NMR Spectrum of complex **B** in d_8-THF

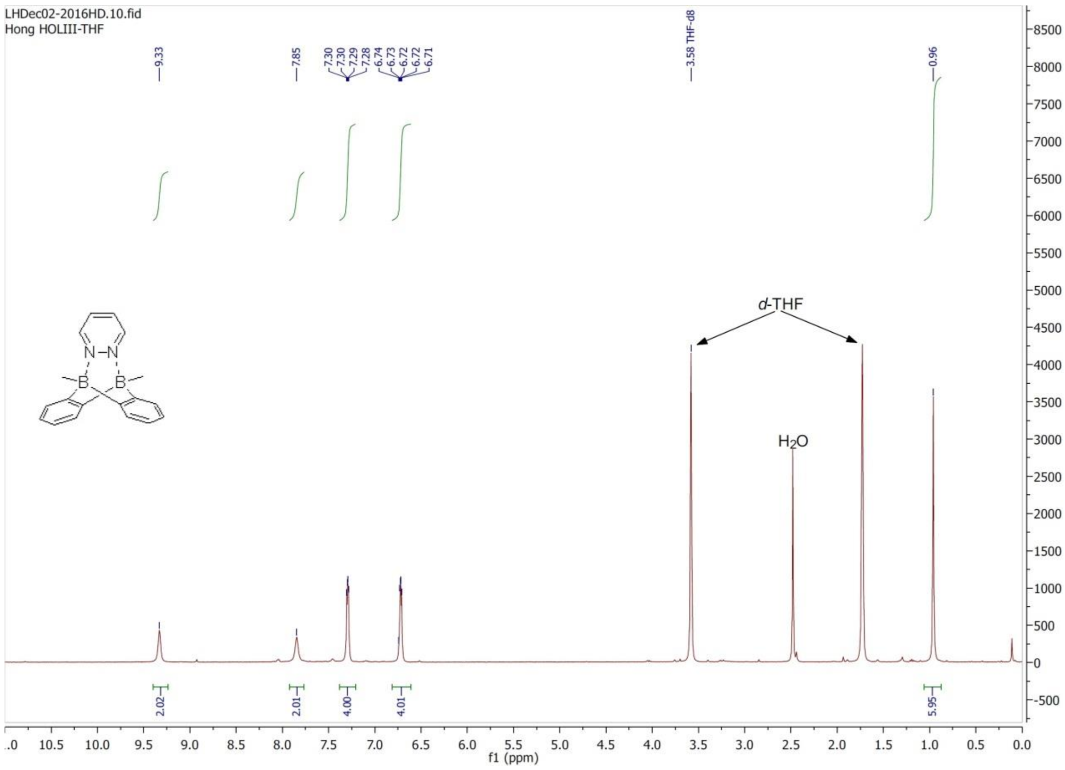

^{13}C NMR Spectrum of complex **B** in d_8-THF

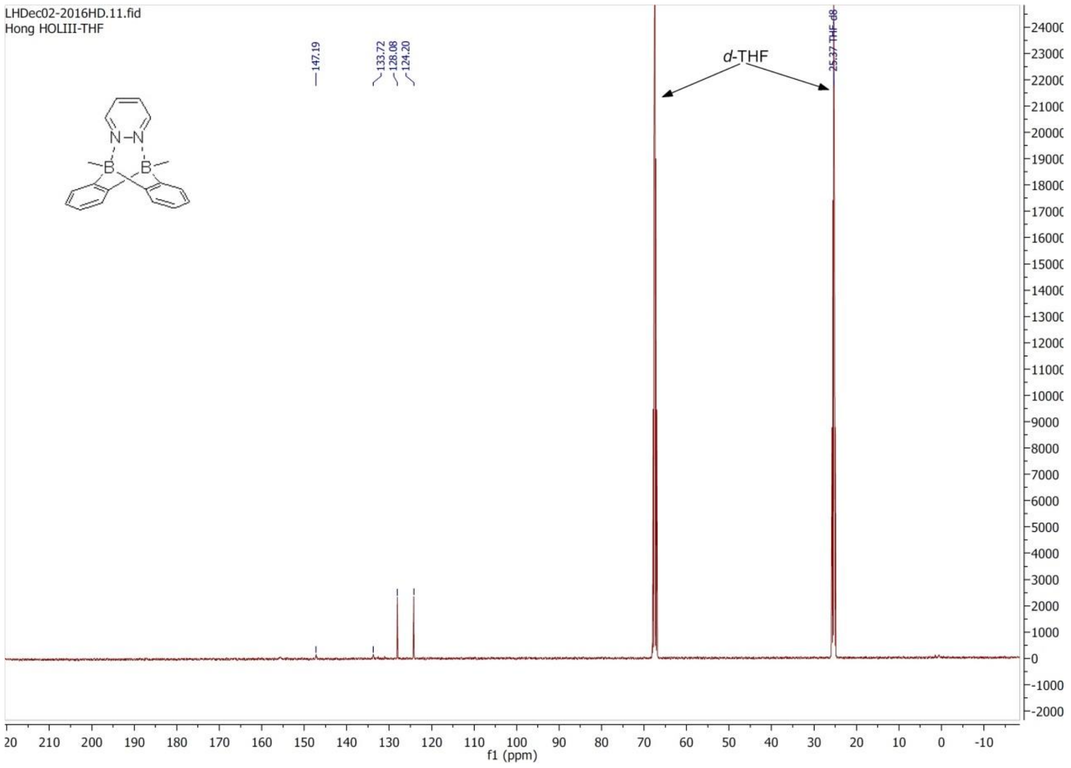

^{11}B NMR Spectrum of complex B in d_8-THF

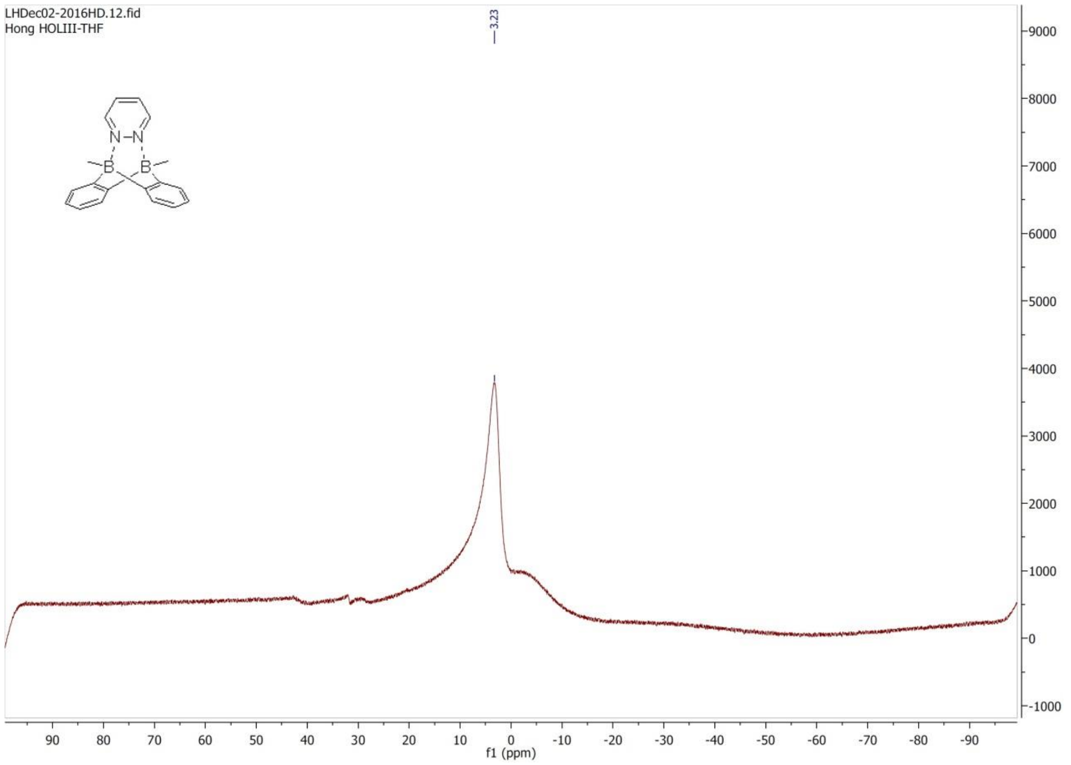

IEDDA reactions catalyzed by the air-stable bidentate Lewis acid catalyst B

General procedure A for IEDDA reactions of phthalazine: In a Schlenk tube charged with a stirring bar, the air-stable bidentate Lewis acid catalyst **B** (5.00 mol %) and the stated solvent were added under N_2. Then, the phthalazine (1.00 equiv), dienophile (2.00 equiv; for enamines, generated in situ from aldehyde and amine) were added subsequently. The reaction mixture was stirred at the given temperature. After the reaction was finished, the solvent was removed. The remaining residue was purified by flash column chromatography over SiO_2 to obtain the product.

General procedure B for IEDDA reactions of 1,2,4,5-tetrazine: The air-stable bidentate Lewis acid catalyst **B** (25.0 µmol, 5.00 mol %) and 1,2,4,5-tetrazine (5.00 equiv) in CF_3Ph (2.5 mL) were thoroughly stirred for several minutes. Then, 1,4-naphthoquinone (1.00 equiv) was added, the reaction mixture was heated at 110 °C for 20 h. The solvent, together with the excess of 1,2,4,5-tetrazine were distilled off from the resulting mixture in vacuo. The residue was purified by column chromatography over SiO_2 (ethylacetate/cyclohexane 1:1) to obtain the product.

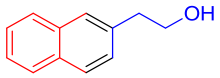

2-(Naphthalen-2-yl)ethanol (**5a**) **[1]**

According to the general procedure (*A*), the catalyst **B** (7.1 mg, 25.0 µmol, 5.00 mol %), phthalazine (**3**, 66.4 mg, 0.500 mmol, 1.00 equiv), diglyme (0.6 mL), 2,3-dihydrofuran (**4a**, 70.1 mg, 75.6 µl, 1.00 mmol, 2.00 equiv) and *N*,*N*-diisopropylethylamine (200 µl) were stirred for 3 d at 170 °C. After evaporation of diglyme at 80 °C/10 mbar, the residue was purified by flash column chromatography to obtain the naphthalene **5a** (14.6 mg, 17%). **^{1}H NMR** ($CDCl_3$, 400 MHz): δ 7.83 – 7.79 (m, 3H), 7.69 (s, 1H), 7.50 – 7.43 (m, 2H), 7.37 (dd, ^3J = 8.4 Hz, ^4J = 1.8, 1H), 3.95 (t, ^3J = 6.5 Hz, 2H), 3.04 (t, ^3J = 6.5 Hz, 2H), 1.49 (br, 1H). Spectroscopic data for the title compound was consistent with the literature.

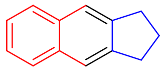

2,3-Dihydro-1*H*-cyclopenta[*b*]naphthalene (5b) **[1]**

According to the general procedure (*A*), the catalyst **B** (6.3 mg, 22.0 µmol, 5.00 mol %), phthalazine (**3**, 57.8 mg, 0.440 mmol, 1.00 equiv), diglyme (0.45 mL), cyclopentanone (**4b**, 65.7 µL, 0.735 mmol, 1.67 equiv), pyrrolidine (**4c**, 73.0 µL, 0.880 mmol, 2.00 equiv) were stirred for 60 h at 55 °C. After work-up with *m*CPBA and evaporation of diglyme at 80 °C/10 mbar, the residue was purified by flash column chromatography to yield the naphthalene **5b** (26.0 mg, 35%). **^{1}H NMR** ($CDCl_3$, 400 MHz): δ 7.78 (dd, ^3J = 2.3, 6.1 Hz, 2 H), 7.68 (s, 2H), 7.40 (dd, ^3J = 3.3, 6.2 Hz, 2H), 3.08 (td, ^3J = 7.3 Hz, ^4J = 1.1 Hz, 4H), 2.20 – 2.13 (m, 2H). Spectroscopic data for the title compound was consistent with the literature.

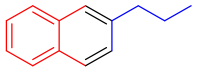

2-Propylnaphthalene (5c) **[7]**

According to the general procedure (*A*), the catalyst **B** (4.4 mg, 15.4 µmol, 5.00 mol %), phthalazine (**3**, 40.4 mg, 0.307 mmol, 1.00 equiv), THF (0.5 mL), pentanal (**4d**, 83.3 µl, 67.5 mg, 0.767 mmol, 2.50 equiv) and pyrrolidine (**4c**, 30.6 µl, 26.5 mg, 0.368 mmol, 1.20 equiv) were stirred for 20 h at 60 °C. The solvent was then removed under reduced pressure, and the residue was purified by flash column chromatography to obtain the naphthalene **5c** (9.0 mg, 17 %). **^{1}H NMR** ($CDCl_3$, 400 MHz): δ 7.85 – 7.74 (m, 3H), 7.61(s, 1H), 7.50 – 7.38 (m, 2H), 7.37 – 7.31 (m, 1H), 2.88 – 2.63 (m, 2H), 1.89 – 1.64 (m, 2H), 0.99 (t, ^3J = 7.4 Hz, 3H). Spectroscopic data for the title compound was consistent with the literature.

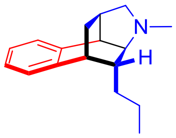

1,3,3a,4,5,9b-Hexahydro-2-methyl-10-propyl-(1*R*,3a*S*,5*S*,9b*S*,10*S*)-*rel*-1,5-methano-2*H*-benz[*e*]isoindole (**5d**) **[8]**

According to the general procedure (*A*), catalyst **B** (8.4 mg, 29.6 μmol, 5.00 mol %), phthalazine (**3**, 77.8 mg, 0.592 mmol, 1.00 equiv), THF (1.5 mL), pentanal (**4d**, 159 μl, 129 mg, 1.47 mmol, 2.48 equiv) and *N*-allylmethylamine (**4e**, 70.4 μl, 52.2 mg, 0.704 mmol, 1.19 equiv) were stirred for 15 h at 60 °C. The solvent was then removed under reduced pressure, and the residue was purified by flash column chromatography to obtain the naphthalene **3d** (68.7 mg, 48 %). **^{1}H NMR** ($CDCl_3$, 400 MHz): δ 7.22 – 7.17 (m, 1H), 7.19 – 7.17 (m, 2H), 7.12 – 7.08 (m, 1H), 3.25 (t, ^3J = 4.4, 1H), 3.07 (dd, ^3J = 5.4, 9.6 Hz, 1H), 2.81 (q, ^3J = 3.0 Hz, 1H), 2.62 (d, ^3J = 9.6 Hz, 1H), 2.51 (s, 3H), 2.46 (d, ^3J = 4.4 Hz, 1H), 2.20 – 2.12 (m, 1H), 1.84 (td, ^3J = 3.2, 7.7 Hz, 1H), 1.69 (ddd, ^3J = 2.2, 10.0, 12.4 Hz, 1H), 1.58 – 1.50 (m, 1H), 1.32 – 1.25 (m, 2H), 0.79 (t, ^3J = 7.4 Hz, 3H), 0.75 – 0.64 (m, 1H), 0.62 – 0.49 (m, 1H). Spectroscopic data for the title compound was consistent with the literature.

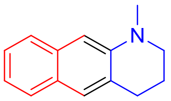

1-Methyl-1,2,3,4-tetrahydrobenzo[*g*]quinoline (**5e**) **[1]**

According to the general procedure (*A*), catalyst **B** (8.4 mg, 29.6 μmol, 5.00 mol %), phthalazine (**3**, 77.8 mg, 0.592 mmol, 1.00 equiv), CF_3Ph (1.5 mL), 6-ethoxy-1-methyl-1,2,3,4-tetrahydropyridine (**4f**, 167 mg, 1.18 mmol, 2.00 equiv) were stirred for 19 h at 100 °C. The solvent was then removed under reduced pressure and the residue was purified by flash column chromatography to obtain the naphthalene **5e** (107 mg, 92 %). **^{1}H NMR** ($CDCl_3$, 400 MHz): δ 7.69 – 7.61 (m, 2 H), 7.44 (s, 1 H), 7.38 – 7.32 (m, 1 H), 7.23 – 7.17 (m, 1 H), 6.82 (s, 1 H), 3.37 (t, ^3J = 5.9

Hz, 2 H), 3.05 (s, 3 H), 2.98 (t, ^{3}J = 6.3 Hz, 2 H), 2.11 – 2.02 (m, 2 H). Spectroscopic data for the title compound was consistent with the literature.

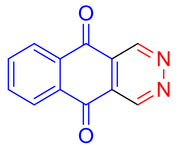

2,3-Diaza-9,10-anthraquinone (**8a**) **[9]**

According to the general procedure (*B*), catalyst **B** (2.8 mg, 10.0 µmol, 5.00 mol %), 1,4-naphthaquinone (**7a**, 31.6 mg, 0.200 mmol, 1.00 equiv), tetrazine (**6**, 82.1 mg, 1.00 mmol, 5.00 equiv) and CF_3Ph (1.0 mL) were stirred for 20 h at 110 °C. The solvent was then removed under reduced pressure and the residue was purified by flash column chromatography to give the pure product (39.0 mg, 93 %). **^{1}H NMR** ($CDCl_3$, 400 MHz): δ 10.00 (s, 2H), 8.33 (dd, ^{3}J = 4.0, 4.0 Hz, 2H), 7.94 (dd, ^{3}J = 4.0, 4.0 Hz, 2H). Spectroscopic data for the title compound was consistent with the literature.

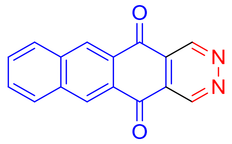

2,3-Diaza-5,12-naphthacenedione (**8b**) **[9]**

According to the general procedure (*B*), catalyst **B** (2.8 mg, 10.0 µmol, 5.00 mol %), anthracene-1,4-dione (**7b**, 41.6 mg, 0.200 mmol, 1.00 equiv), tetrazine (**6**, 82.1 mg, 1.00 mmol, 5.00 equiv) and CF_3Ph (1.0 mL) were stirred for 20 h at 110 °C. The solvent was then removed under reduced pressure and the residue was purified by flash column chromatography to give the pure product (42.7 mg, 82 %). **^{1}H NMR** (CF_3CO_2D, 400 MHz) δ 10.26 (d, ^{5}J = 2.6 Hz, 2H), 8.94 (d, ^{5}J = 2.8 Hz, 2H), 8.11 – 8.14 (m, 2H), 7.78 – 7.81 (m, 2H). Spectroscopic data for the title compound was consistent with the literature.

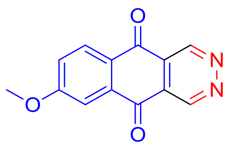

6-Methoxy-2,3-diaza-9,10-anthraquinone (**8c**)

According to the general procedure (*B*), catalyst **B** (4.0 mg, 14.0 µmol, 5.00 mol %), 6-methoxy-1,4-naphthaquinone (**7c**, 52.7 mg, 0.280 mmol, 1.00 equiv), tetrazine (**6**, 115 mg, 1.40 mmol, 5.00 equiv) and CF_3Ph (3.0 mL) were stirred for 20 h at 110 °C. The solvent was then removed under reduced pressure and the residue was purified by flash column chromatography to give the pure product (59.3 mg, 88 %). **^{1}H NMR** ($CDCl_3$, 400 MHz): δ 9.99 (d, ^5J =1.2 Hz, 1H), 9.95 (d, ^5J = 1.2 Hz, 1H), 8.27 (d, ^3J = 8.7 Hz, 1H), 7.71 (d, ^4J = 2.6 Hz, 1H), 7.38 (dd, ^3J = 8.7 Hz, ^4J = 2.6 Hz, 1H), 4.02 (s, 3H); **^{13}C NMR** (100 MHz, $CDCl_3$): δ 182.6, 181.0, 165.6, 147.4, 146.9, 134.6, 130.3, 126.1, 125.63, 125.59, 122.6, 110.5, 56.4; **HRMS (ESI)** m/z calcd for $C_{13}H_8N_2O_3$ $[M+Na]^+$ 263.0427, found: 263.0429; **Mp:** 178.3 °C–178.7 °C.

^{1}H NMR Spectrum of 6-methoxy-2,3-diaza-9,10-anthraquinone in $CDCl_3$ (**8c**)

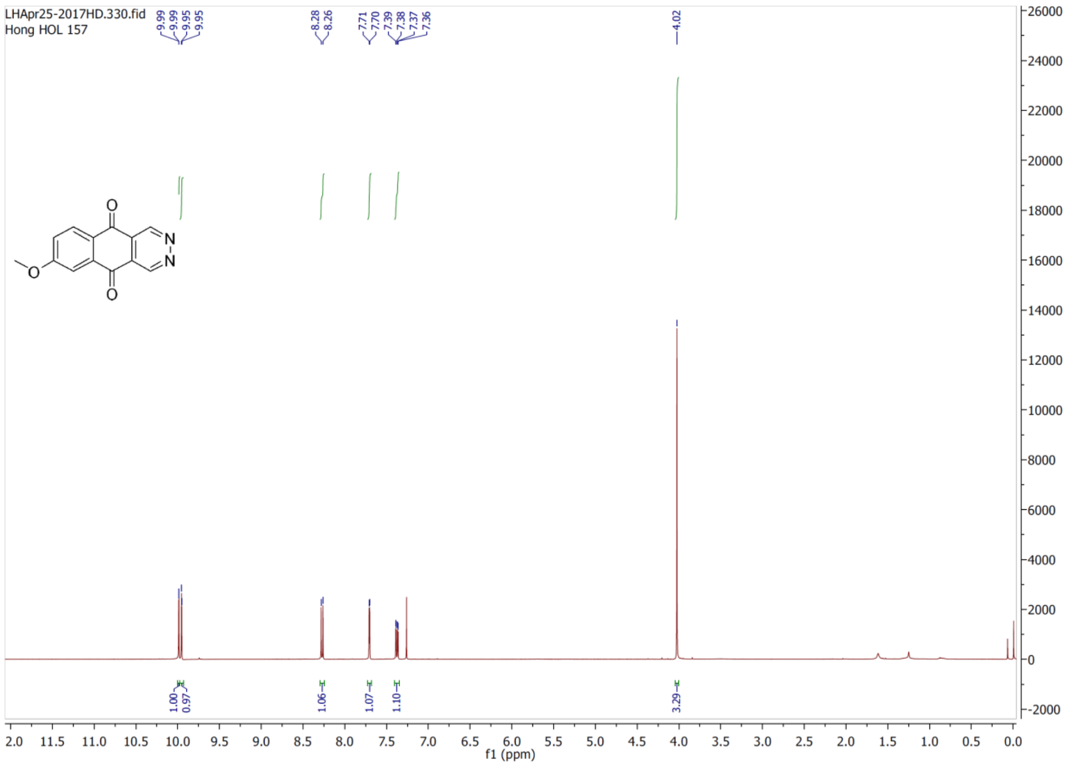

^{13}C NMR Spectrum of 6-methoxy-2,3-diaza-9,10-anthraquinone in $CDCl_3$ (**8c**)

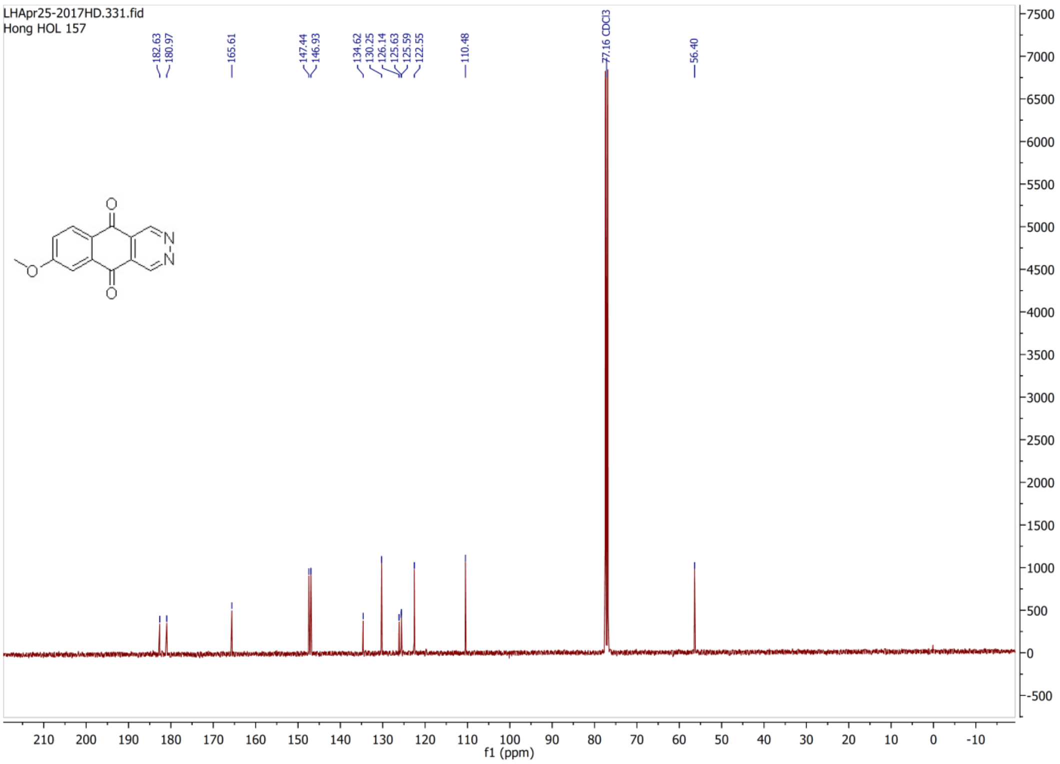

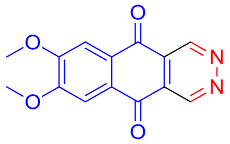

6,7-Dimethoxy-2,3-diaza-9,10-anthraquinone (**8d**)

According to the general procedure (*B*), catalyst **B** (4.0 mg, 14.0 μmol, 5.00 mol %), 6,7-dimethoxy-1,4-naphthaquinone (**7d**, 61.1 mg, 0.280 mmol, 1.00 equiv), tetrazine (**6**, 115 mg, 1.40 mmol, 5.00 equiv) and CF_3Ph (3.0 mL) were stirred for 20 h at 110 °C. The solvent was then removed under reduced pressure and the residue was purified by flash column chromatography to give the pure product (71.9 mg, 95 %). **^{1}H NMR** ($CDCl_3$, 400 MHz): δ 9.95 (s, 2H), 7.69 (s, 2H), 4.10 (s, 6H); **^{13}C NMR** (100 MHz, $CDCl_3$): δ 181.6, 155.2, 147.0, 127.7, 125.6, 108.4, 56.9; **HRMS (ESI)** m/z calcd for $C_{14}H_{10}N_2O_4$ $[M+Na]^+$ 293.0533, found: 293.0527; **Mp:** 243.7 °C–244.9 °C.

^{1}H NMR Spectrum of 6,7-dimethoxy-2,3-diaza-9,10-anthraquinone in $CDCl_3$ (**8d**)

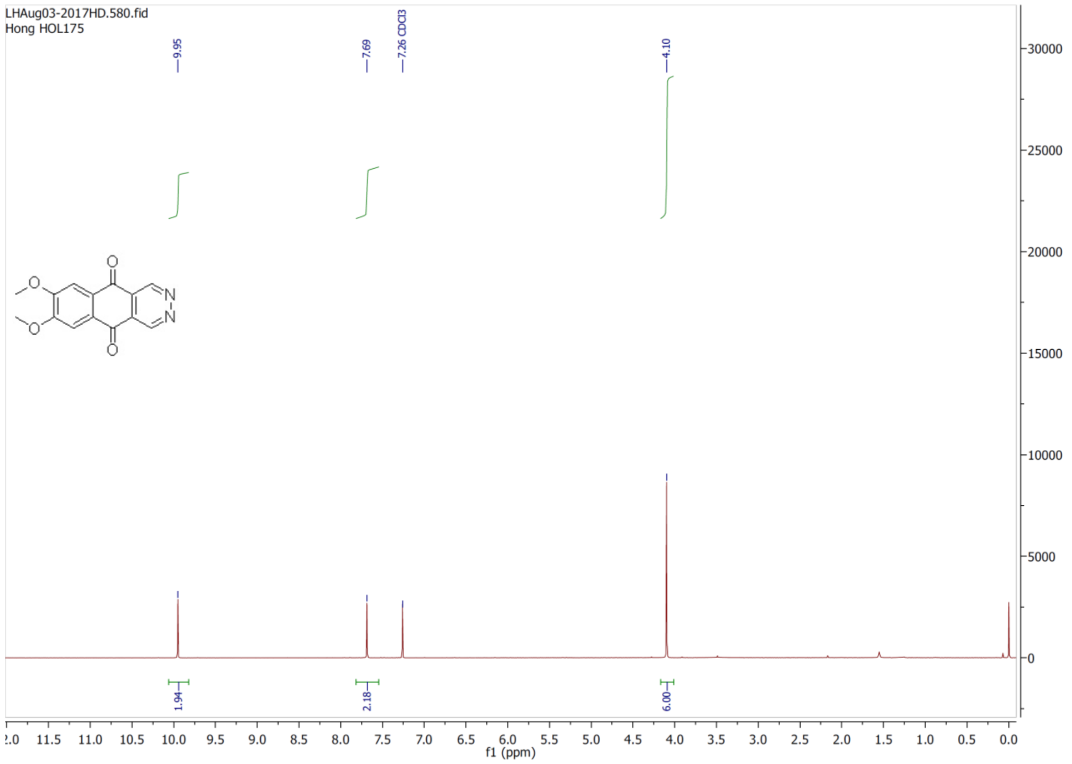

^{13}C NMR Spectrum of 6,7-dimethoxy-2,3-diaza-9,10-anthraquinone in $CDCl_3$ (**8d**)

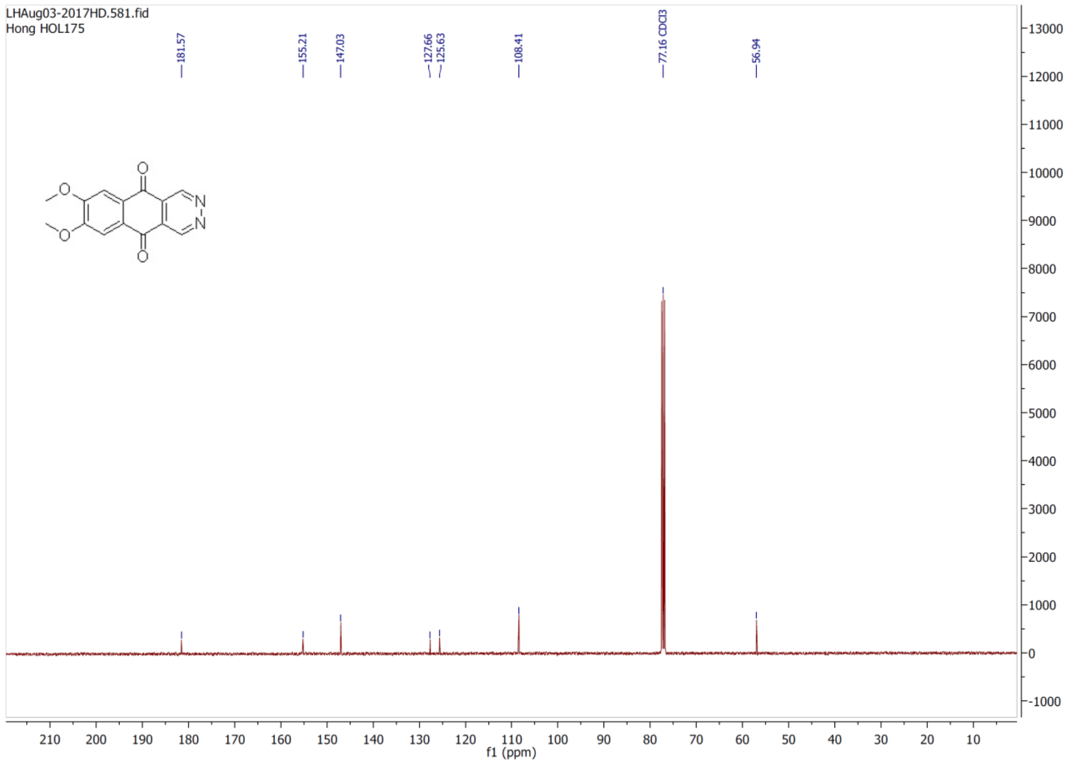

UV–vis spectra

UV–vis spectrum of adduct complex **B** in $CHCl_3$ (concentration: 4.52×10^{-5} mol/L)

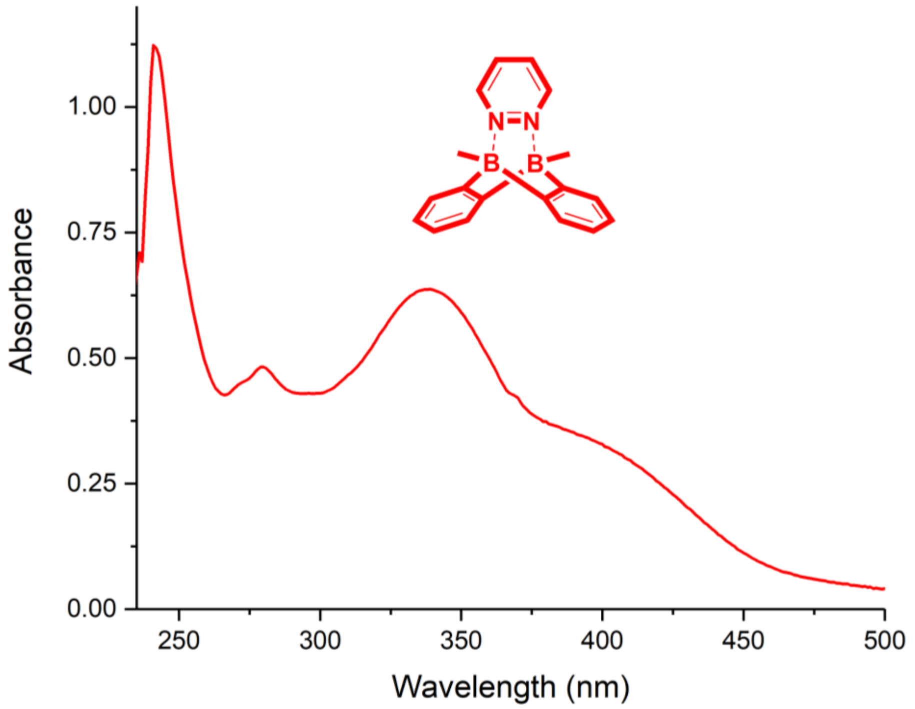

UV–vis spectrum of bisborane **A** in $CHCl_3$ (Concentration: 4.52×10^{-5} mol/L)

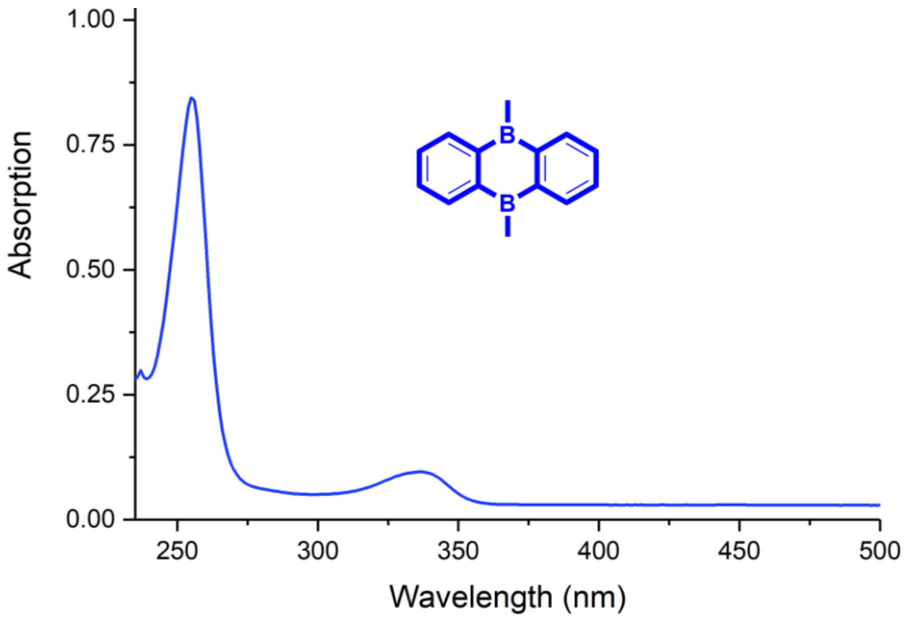

UV–vis spectrum of pyridazine in $CHCl_3$ (concentrations: 3.68×10^{-5} mol/L)

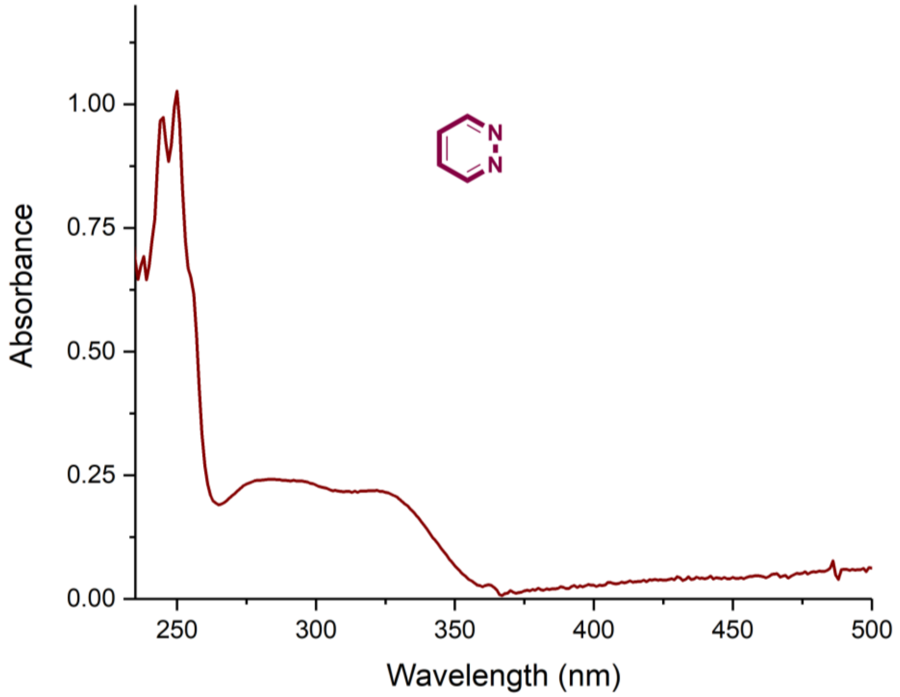

X-ray crystallography

Some single crystals of $C_{18}H_{18}B_2N_2$ * 0.5 ($C_4H_8O_2$) * 0.17 ($C_6H_5Br_1$) were transferred into inert oil (Fomblin Y, 1600 cst, Sigma Aldrich GmbH, Steinheim, Germany). A suitable crystal was then mounted onto a micromount sample holder (MiTeGen, Dual-Thickness MicroMounts, 100 µm) and immediately placed into a stream of cold N_2 (100K) inside the diffractometer (Bruker D8 Venture, Bruker, Karlsruhe, Germany). MoKα radiation (λ = 71.073 pm) from an Incoatec microsource was used. After unit cell determination, the reflection intensities were collected. The software of the diffractometer (Bruker Apex III)[10] was used for data collection, unit cell determination and processing of the raw data. Absorption correction was applied using SADABS [11]. The structure was solved by Direct Methods using SHELXS [12], full matrix least squares refinement on $|F^2|$ using SHELXL-2016/6 [12] as implemented in the Olex2- program [13] was used for structure refinement.

All non-hydrogen atoms could be refined with anisotropic displacement parameters. EADP constraints were used for the refinement of some of the ethylacetate molecules. All hydrogen atoms could be refined using AFIX codes of SHELXL. The program Diamond was used for graphical representations [14]. The CCDC reference number is 1567302.

Table S1: Crystal data and structure refinement data of compound $C_{18}H_{18}B_2N_2$ * 0.5 ($C_4H_8O_2$) * 0.17 ($C_6H_5Br_1$) for the X-ray measurement at 100 K.

Empirical formula	$C_{18}H_{18}B_2N_2$ * 0.5 ($C_4H_8O_2$) * 0.17 ($C_6H_5Br_1$)
Formula weight	354.10 g/ mol
Temperature	100 K
Wavelength	71.073 pm
Crystal system	monoclinic
Space group	$P2_1/n$
Unit cell dimensions	a = 33.8537(14 Å
	b = 13.8270(6) Å
	c = 24.6202(10) Å
	β = 90.773(1)°
Volume	V = 11523.5(8) x 10^6 $Å^3$
No. of formula units	24
Density (calculated)	1.225 g/cm^3
F(000)	4478
Theta range for data collection	2.29 to 27.90°
Index ranges	-44<=h<=44, -18<=k<=18, -31<=l<=32
R_{int} and R_σ	0.0775 and 0.0407
Reflections collected	425631
Independent reflections	27661
Crystal size	0.137 x 0.147 x 0.244 mm^3
Absorption coefficient	0.418 mm^{-1}
Absorption correction	numerical
Max. and min. transmission	0.945 and 0.905
Refinement method	Full-matrix least-squares on F^2
Data/ restraints/ parameters	27661 / 0 / 1417
Goodness-of-fit on F^2	1.028
Final R- indices (I>2sigma(I)	R_1 = 0.0775, wR_2 = 0.1935
R indices (all data)	R_1 = 0.01105, wR_2 = 0.2148
Largest diff. peak and hole	3.097 and -1.244 $e^-/Å^3$

References:

1 Kessler, S. N.; Neuburger, M.; Wegner, H. A. *Eur. J. Org. Chem.*, **2011**, 3238–3245.

2 Jiang, Y.; Li, X.; Zhao, Y.; Jia, S.; Li, M.; Zhao, Z.; Zhang, R.; Li, W.; Zhang, W. *RSC Advances* **2016**, *6*, 110102–110107.

3 Wiley, R. H. *J. Macromol. Sci. Part A-Pure Appl. Chem.* **1987**, *24*, 1183–1190.

4 Nor, S. M.; Sukari, M. A.; Azziz, S. S.; Fah, W. C.; Alimon, H.; Juhan, S. F. *Molecules* **2013**, *18*, 8046–8062.

5 Zeng, B.-B.; Ren, J.; Lu, L.; Xu, J.; Yu, T. *Synthesis* **2015**, *47*, 2270–2280.

6 Domasevitch, K. V.; Gural'skiy, I. A.; Solntsev, P. V.; Rusanov, E. B.; Krautscheid, H.; Howard, J. A.; Chernega, A. N. *Dalton Trans.* **2007**, 3140–3148.

7 Schweighauser, L.; Bodoky, I.; Kessler, S. N.; Häussinger, D.; Wegner, H. A. *Synthesis* **2012**, *44*, 2195–2199.

8 Schweighauser, L.; Bodoky, I.; Kessler, S. N.; Häussinger, D.; Donsbach, C.; Wegner, H. A. *Org. Lett.* **2016**, *18*, 1330–1333.

9 Hong, L.; Ahles, S.; Strauss, M. A.; Logemann, C.; Wegner, H. A. *Org. Chem. Front.* **2017**, *4*, 871–875.

10 *Bruker Apex 3 (v2015.5-2)*, Bruker AXS, Karlsruhe, Germany, **2015**.

11 *SADABS-2014/4, Program for Area Detector Absorption Correction*, G. M. Sheldrick: Göttingen, **2014**.

12 G. Sheldrick, *Acta Crystallogr.* **2015**, *C71*, 3–8.

13 O. V. Dolomanov, L. J. Bourhis, R. J. Gildea, J. A. K. Howard, H. Puschmann, *J. Appl. Crystallogr.* **2009**, *42*, 339–341.

14 *Diamond 4.1.2*, K. Brandenburg: Bonn, **2016**.
